# Musculoskeletal disorders among office workers: prevalence, ergonomic risk factors, and their interrelationships

**DOI:** 10.1038/s41598-025-30155-6

**Published:** 2025-11-26

**Authors:** Mostafa Mohammadian, Sina Mollahoseini, Ahmad Naghibzadeh-Tahami

**Affiliations:** 1 Health Foresight and Innovation Research Center, Institute for Futures Studies in Health, Kerman University of Medical Sciences, Haft Bagh-e-Alavi Road, Kerman, Iran; 2 HSE Specialist at Golgohar Mining and Industrial Company, Sirjan, Iran; 3 Health Services Management Research Center, Institute for Futures Studies in Health, Kerman University of Medical Sciences, Kerman, Iran

**Keywords:** Musculoskeletal disorders, Back pain, Posture, Office workers, Ergonomic risk factors, Diseases, Health care, Health occupations, Medical research, Risk factors

## Abstract

This cross-sectional study investigated the prevalence of work-related musculoskeletal disorders (WMSDs), ergonomic risks, and psychosocial factors among 99 office workers at an industrial company. Participants, aged 20–50 years with minimum one year of experience, were selected using census sampling. Exclusion criteria comprised history of musculoskeletal disorders, fractures, hand surgery, major systemic diseases, pregnancy, menstruation, or recent hospitalization. Data collection utilized the extended nordic musculoskeletal questionnaire and rapid office strain assessment (ROSA). Results demonstrated 80.81% WMSDs prevalence, most commonly affecting neck (58.6%), lower back (52.5%), and shoulders (37.4%). The mean ROSA score of 5.40 ± 1.27 indicated suboptimal workstation ergonomics. Significant associations were identified between chair height and knee/shoulder/upper back pain (*p* < 0.01), and between job stress and WMSDs across all body regions (*p* < 0.05). The findings emphasize the necessity of comprehensive interventions targeting ergonomic improvements, postural correction, and stress management to mitigate WMSDs risks in office environments.

**Supplementary Information:**

The online version contains supplementary material available at 10.1038/s41598-025-30155-6.

## Introduction

Work-related musculoskeletal disorders (WMSDs) are among the most common occupational illnesses and injuries^1–3^. These disorders account for roughly 40% of total occupational compensation costs in developed countries^4^. Studies estimate that the direct and indirect costs of musculoskeletal disorders amount to approximately 1% of the gross national product in industrialized nations^5^. WMSDs involve damage to components of the musculoskeletal system—including muscles, peripheral nerves, joints, bones, tendons, ligaments, and blood vessels—caused by improper use or prolonged overexertion^6^. Symptoms typically begin with fatigue, pain, and discomfort, eventually progressing into chronic musculoskeletal conditions that impair limb mobility and reduce muscle strength and function^7^.

Numerous risk factors contribute to the development of these disorders, which can be categorized into physical, psychological, organizational, and individual factors^4^. From an ergonomic standpoint, the most significant risks include prolonged awkward postures, repetitive movements, static exertions, and non-ergonomic workstation design—all of which are prevalent in office environments^8^.With the advancement of information technology and evolving work patterns, computer usage time in office settings has risen significantly, further increasing the risk of musculoskeletal disorders^9^. These conditions not only reduce productivity and increase absenteeism but also severely diminish employees’ occupational quality of life^10,11^.

Improper sitting postures in office workstations constitute one of the most significant risk factors for developing musculoskeletal disorders (MSDs), particularly in the lumbar and spinal regions^12^. Common office postures such as forward bending or torso twisting increase compressive forces on intervertebral discs and surrounding soft tissues, heightening the long-term risk of spinal injuries^13^. Moreover, the static nature of sitting reduces local blood circulation and promotes the accumulation of muscle tension. Without adequate recovery, this may lead to chronic fatigue and an increased likelihood of musculoskeletal abnormalities^14^. To effectively assess the contribution of individual MSD risk factors, validated evaluation and quantification tools are essential. Among various ergonomic assessment methods, the Rapid Office Strain Assessment (ROSA) has emerged as a reliable and widely adopted tool for office environments^15–17^. This visually based checklist method enables rapid yet systematic evaluation of office workstations^15^.

As established, epidemiological surveillance of MSDs and ergonomic workstation assessments are critical for identifying high-risk patterns and developing targeted interventions. These evaluations not only reduce MSD prevalence but also substantially improve workforce productivity and occupational health through enhanced working conditions^18^.Research demonstrates a complex interaction between biomechanical factors (e.g., awkward postures), psychological factors (e.g., job stress), and organizational/individual factors (e.g., physical activity levels and work shift duration) in both the development and persistence of these disorders^19–21^. Accordingly, the present study was designed to investigate WMSD prevalence, identify major ergonomic risk factors in workstations (including postural stressors, psychological stress, and demographic variables), and analyze their associations among office workers within an industrial company in Iran. The study was conducted in a large mining and industrial company where, beyond core production activities, comprehensive regional development operations including urban planning and infrastructure management are carried out. This unique context creates distinct ergonomic challenges at the interface of administrative and industrial functions. Understanding WMSD patterns in such integrated industrial-administrative environments can provide valuable insights for developing targeted interventions.

## Methods

### Participants and study design

**Fig. 1:**
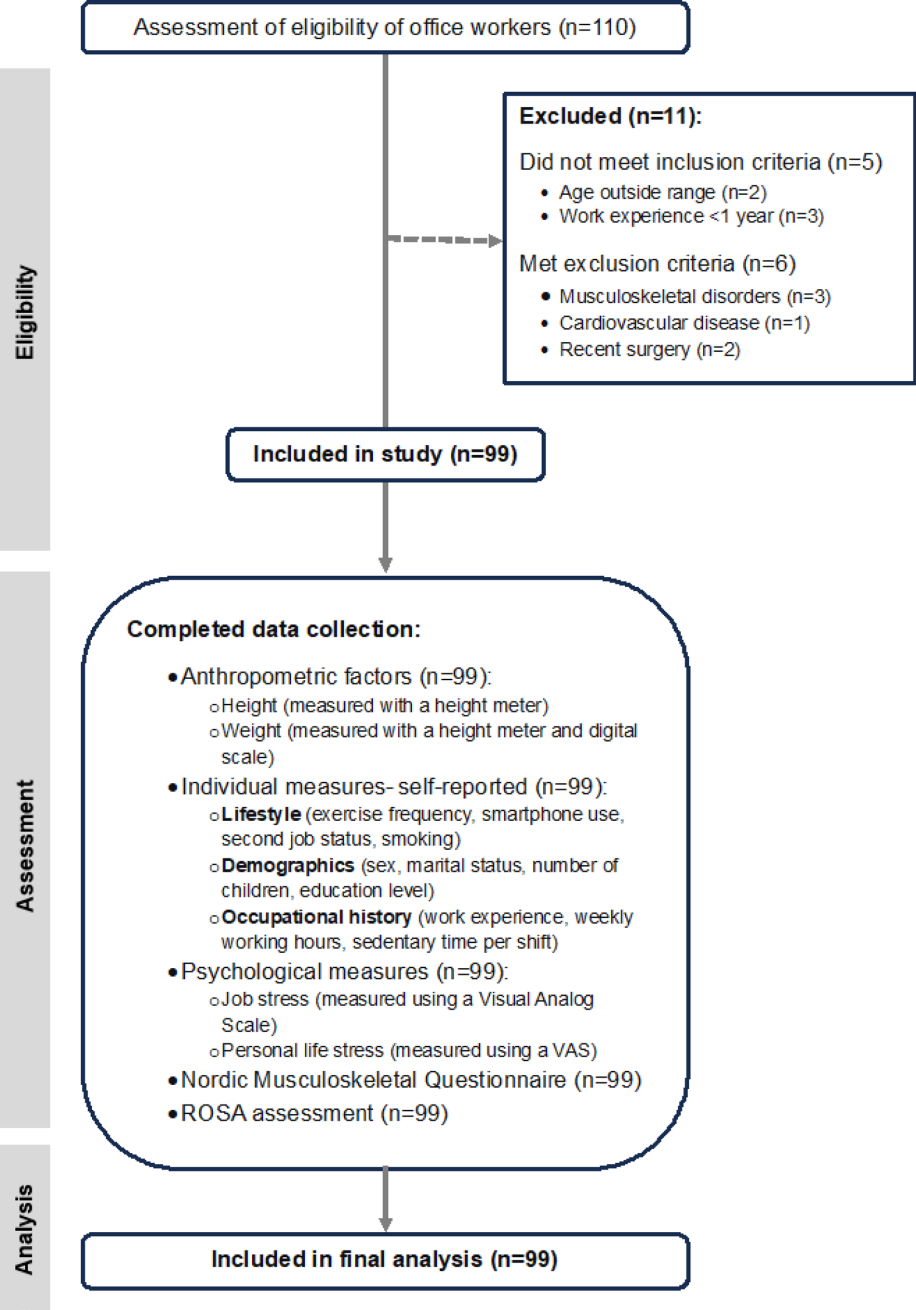
Participant recruitment STROB^*^ flow chart. ^*^strengthening the reporting of observational studies in epidemiology.

This cross-sectional descriptive-analytical study included 99 office employees from an industrial company, selected through census sampling. The participant recruitment process is detailed in Fig. 1. The inclusion criteria were being in the age range of 20 to 50 years and having at least 1 year of work experience. Exclusion criteria included Pain due to hand surgery, fractures, or any musculoskeletal disorders (e.g., scoliosis, osteoporosis), Psychiatric, cardiovascular, neurological, or metabolic diseases, being in menstruation (to avoid bias in self-reported musculoskeletal symptoms), pregnancy, and hospitalization within 6 months prior to the study.

This study evaluated the prevalence of musculoskeletal disorder symptoms among employees using the Nordic Musculoskeletal Questionnaire-Extended version (NMQ-E). Postural risk levels during office workstation tasks were also assessed via the ROSA method. Given that MSDs are multifactorial—influenced by biomechanical, psychological, and individual factors^22,23^—we sought to provide a rapid estimation of each factor’s contribution to clarify their respective impacts on MSDs prevalence. In addition to ROSA-derived postural assessments, the following measures were collected via self-reporting:

- Psychological factors: *Job stress and Personal life stress* (marital/child relationships, future-related anxieties).
- Individual factors: anthropometrics (height, weight, body mass index [BMI]); demographics (sex, marital status, number of children, education level); Occupational history (work experience, weekly working hours, sedentary time per shift) and lifestyle (exercise frequency, smartphone use at work/home, second job status, smoking/waterpipe use).

The association between these factors and MSDs symptoms in the five most affected body regions (per NMQ-E data) was statistically analyzed.

### Ethical compliance

This study was performed in line with the principles of the Declaration of Helsinki. Approval was granted by the Research Ethics Committee of Kerman University of Medical Sciences (Approval ID: IR.KMU.REC.1404.298). Informed consent was obtained from all individual participants included in the study.

## Tools and instruments

### Nordic musculoskeletal questionnaire-extended version

In this study, the Persian version of the NMQ-E was used to assess MSD symptoms. Evidence from the literature indicates that this version has excellent test-retest reliability for different body regions (kappa coefficients = 0.87–1.00) and strong construct validity (*p* < 0.001) in multiple occupational settings. Its quantitative variables have also shown high reproducibility, with intraclass correlation coefficients (ICC) exceeding 0.88^24^. The NMQ-E allows respondents to report perceived pain or MSD symptoms—including current, one-month, and one-year recall—across nine body regions: neck, shoulders, elbows, wrists, upper back, lower back/hips, thighs, knees, and ankles/feet, visualized via an anatomical body map. Additionally, the tool includes questions about the pain/symptom severity, the time duration of symptoms, medical consultations/medication use, and work absenteeism due to pain.

### Rapid office strain assessment

The Persian version of the ROSA checklist was used to evaluate employees’ postures in office workstations. As a screening tool, ROSA helps identify and diagnose ergonomic deficiencies in computer-based tasks. Psychometric properties have been established in previous studies, showing excellent intra-observer (ICC = 0.88–0.91) and good inter-observer reliability (ICC = 0.51–0.91)^16,26^. The Persian version used in this study has demonstrated excellent convergent validity with the RULA checklist (*r* = 0.75, *P* < 0.001) and satisfactory face validity through cultural adaptation^26^. This pen-paper method involves image-assisted posture analysis. After capturing photographs and completing the checklist, scoring charts are used to determine the priority level for corrective actions. The ROSA assessment is conducted using a checklist-based scoring system, evaluating equipment such as chair, monitor, telephone, mouse, and keyboard. The time duration of use for each piece of equipment by the operator is also factored into the section scores. The final score calculation determines the Priority actions priority. Based on the final ROSA score, the method proposes five Action Levels for the workstation, which indicate the need for action and its urgency. Action Levels range from level 0, requiring no action, to level 4, indicating urgent action is needed, and scores of 5 or higher indicate high risk. Priority actions can be determined based on the partial scores for each workstation element^25^.

### Other risk factors for musculoskeletal disorders

#### Psychological factors

*Job stress and Personal life stress* (marital/child relationships, future-related anxieties) were measured quantitatively using a Visual Analog Scale (VAS), ranging from 1 (minimal stress) to 100 (maximum stress), with participants rating their stress levels. Previous studies have documented moderate to good test-retest reliability for the VAS (Spearman’s ρ = 0.60–0.77)^27^; along with strong correlations with pain intensity (ρ = 0.64–0.84)^28^.

#### Individual factors

Anthropometric factors, height and weight, were measured using a wall-mounted stadiometer (Seca 213) and a calibrated digital floor scale (Seca 803), respectively. These instruments are widely used in clinical research and have demonstrated high validity and excellent reliability^29^. A self-report form was developed to collect other individual factors, including demographics (sex, marital status, number of children, education level); Occupational history (work experience, weekly working hours, sedentary time per shift) and lifestyle (exercise frequency, smartphone use at work/home, second job status, smoking/waterpipe use). Participants self-reported this information, which was then recorded by the examiner.

### Procedure

Data were collected daily from 9:00 AM to 2:00 PM between January 28 and March 10, 2024. Data collection was conducted through a structured, multi-stage process at participants’ workstations. First, eligible participants provided informed consent and completed a demographic form alongside self-rated psychological scales (VAS for job and personal stress). Subsequently, the examiner administered the NMQ-E verbally, recording participants’ responses regarding pain location, severity, and impact. Following the questionnaire, participants were asked to assume their typical working postures while performing routine computer tasks. The examiner then conducted the ROSA, systematically observing and photographing the participant’s posture and workstation setup from multiple angles (front, side). These photographs, alongside direct observation, were used to complete the ROSA checklist scoring. Each evaluation required approximately 15 min to complete.

### Statistical analysis

Descriptive statistics were used to summarize qualitative and quantitative variables. The normality of quantitative variables was assessed using the Shapiro-Wilk test. Independent Samples t-tests were employed to examine differences in quantitative variables (including postural risk scores) between dichotomous qualitative variables (such as the presence or absence of musculoskeletal disorders in different body regions). Pearson chi-square test was used to analyze association between musculoskeletal disorders in body regions and qualitative variables. A significance level of 5% (*p* < 0.05) was adopted for all statistical tests. Data analysis was performed using IBM SPSS software (version 22) at a significance level of 0.05.

## Results

### Participant characteristics

The study included 99 office employees (75 males, 24 females) aged 28–50 years (Mean = 34.41, SD = 5.84). Table 1 presents the demographic characteristics of the participants.

**Table 1:** Demographic characteristics of the studied office workers (*n* = 99).

Quantity variables		Mean (SD)	Min -Max	Qualitative variables		N (%)
Age (year)		34.41 (5.84)	24-53	Gender	Male	75 (75.80)
Height (cm)		176.84 (9.39)	150-192		Female	24 (24.20)
Weight (Kg)		84.94 (17.17)	50-125	Marital status	Single	34 (34.34)
Body mass index (kg/m^2^)		26.99(4.24)	19-37		Married	65 (65.66)
Work experience (year)		9.42 (5.18)	2-25	Level of education	Diploma	2 (2.00)
Weekly working (hours)		44.23 (5.39)	40-72		Associate	4 (4.00)
Sedentary work per day (hours)		5.53 (1.64)	1-9		Bachelor	46 (46.50)
Exercise per week (hours)		3.25 (3.64)	0-16		Master	38 (38.40)
Mobile phone use per day (hours)		4.11 (2.06)	1-12		PhD	9 (9.10)
Job stress (1-100 points)		66.36 (24.03)	5-100	Body mass index	Underweight	0 (0.00)
Personal life stress (1-100 points)		41.26 (26.77)	5-100		Normal weight	37 (37.40)
					Overweight	40 (40.40)
					Obese	22 (22.20)
				Smoking	Yes	25 (25.30)
					No	74 (74.70)
				Job position	Employee	59 (59.60)
					Manager	40 (40.40)
				Exercise adherence	yes	49 (49.50)
					no	50 (50.5)
				Having a second Job	Yes	24 (24.20)
					No	75 (75.80)
				Number of children	No children	53 (53.53)
					One	21 (21.21)
					Two	22 (22.23)
					More than two	3 (3.03)
				**Total**		**99 (100)**

### Prevalence of musculoskeletal disorders

**Fig. 2:**
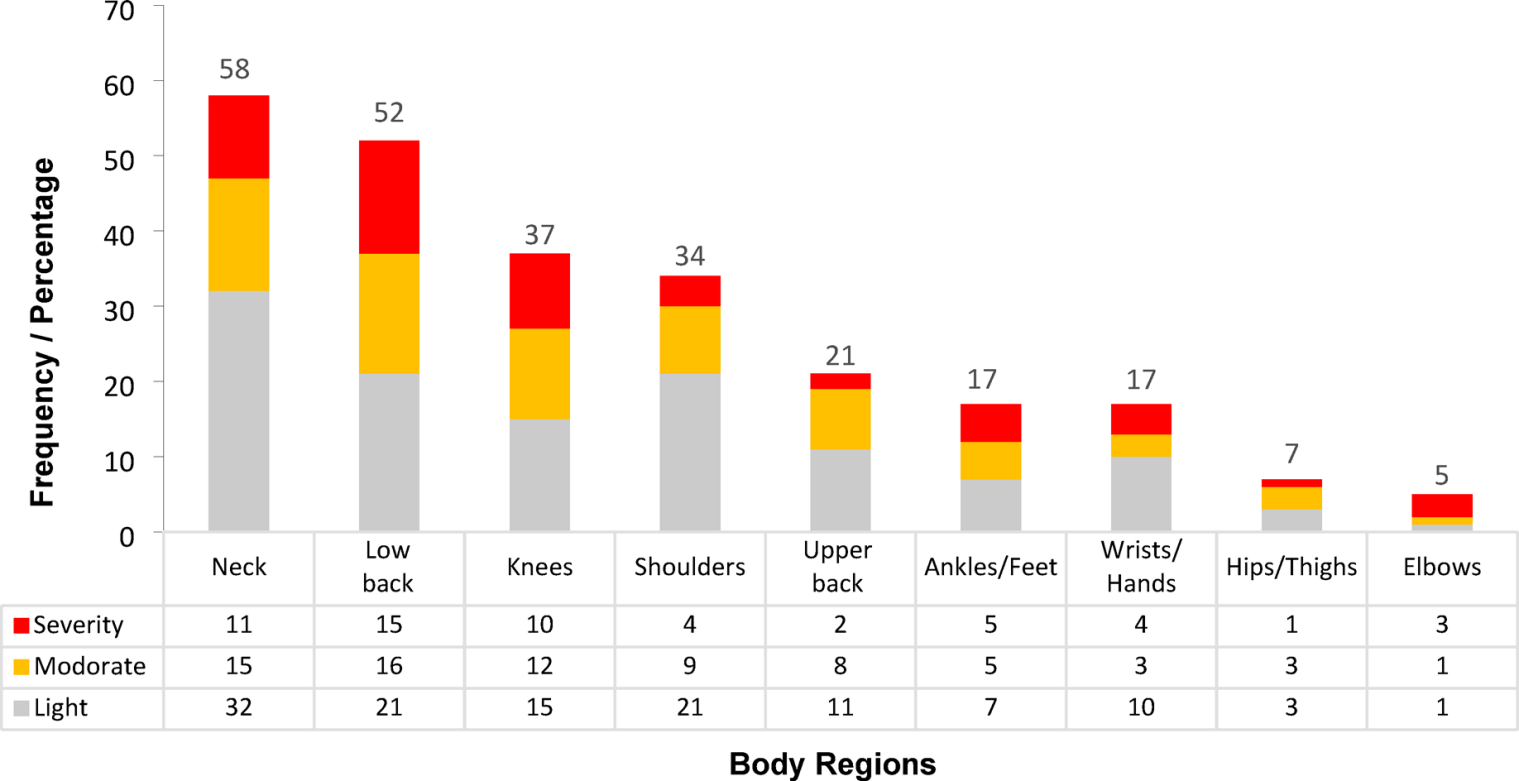
Frequency/percentage of musculoskeletal disorder symptoms occurrence across different body regions by pain severity among studied office workers (*n* = 99). *Due to the sample size approaching 100, the frequency closely approximates the percentage value.

The findings revealed that 80.81% of office workers (*n* = 81) experienced MSDs in at least one body region during the study period, the past month, or the past 12 months. Figure 2 illustrates the frequency (percentage) of MSDs by pain severity. The most commonly affected areas were the neck (58.6%), lower back (52.5%), shoulders (37.4%), knees (34.3%), and upper back (21.2%). Regarding impact on work, 37 workers (37.40%) reported absenteeism due to MSDs-related pain, with lower back pain being the most frequent cause (18.20%), followed by neck pain (14.10%) and knee pain (8.10%). Medical treatment was sought by 20.20%, 19.20%, and 18.20% of participants for lower back, neck, and knee symptoms, respectively. The complete descriptive distribution of responses to the Extended Nordic Questionnaire appears in Appendix 1.

### Ergonomic risk assessment of workstations (ROSA-derived data)

**Fig. 3:**
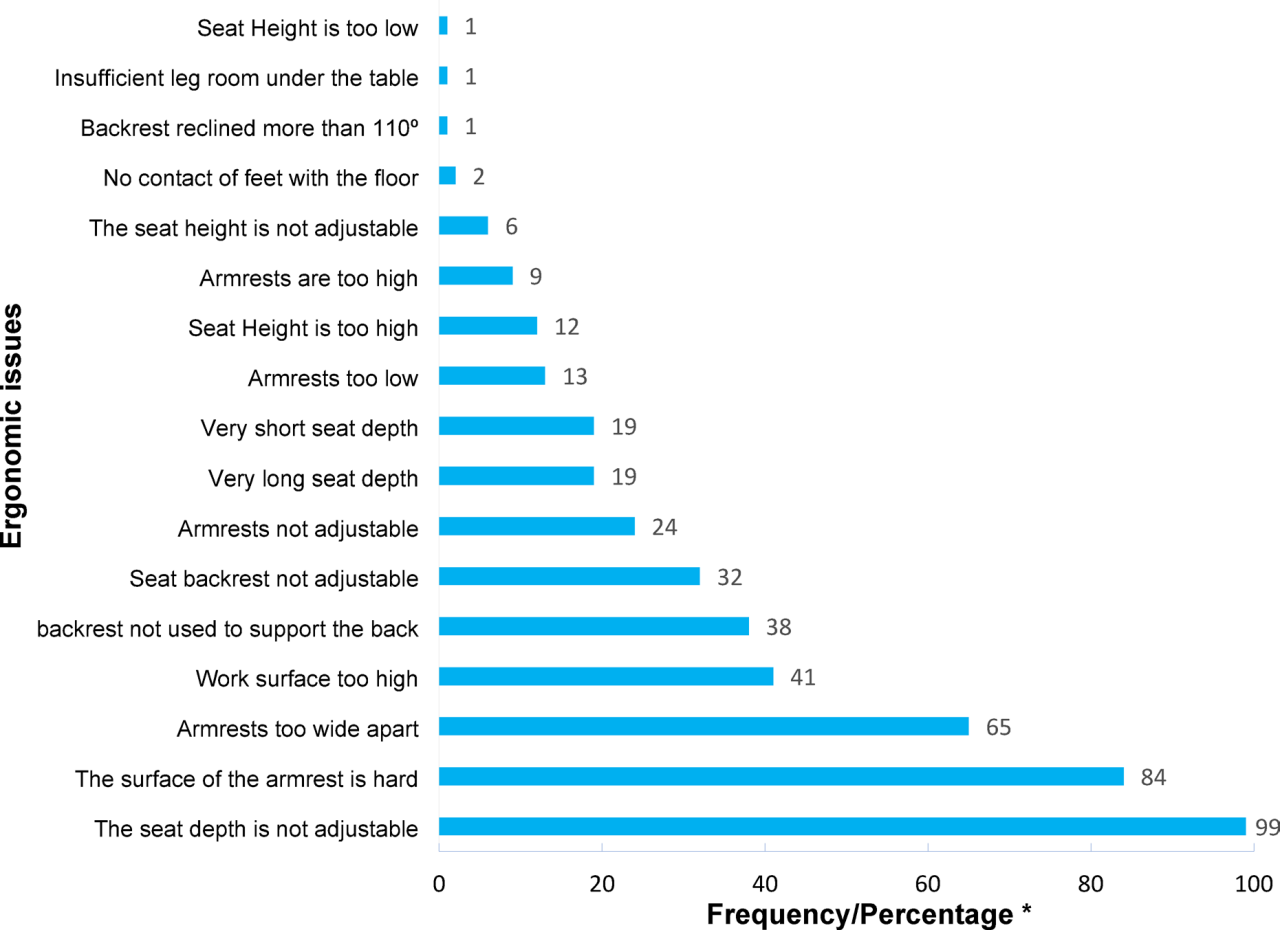
Frequency/percentage of observed ergonomic issues in chairs (including seat height, seat depth, armrests, and backrest components) using the ROSA checklist. *Due to the sample size being close to 100, the frequency is approximately equal to the percentage value.

**Fig. 4:**
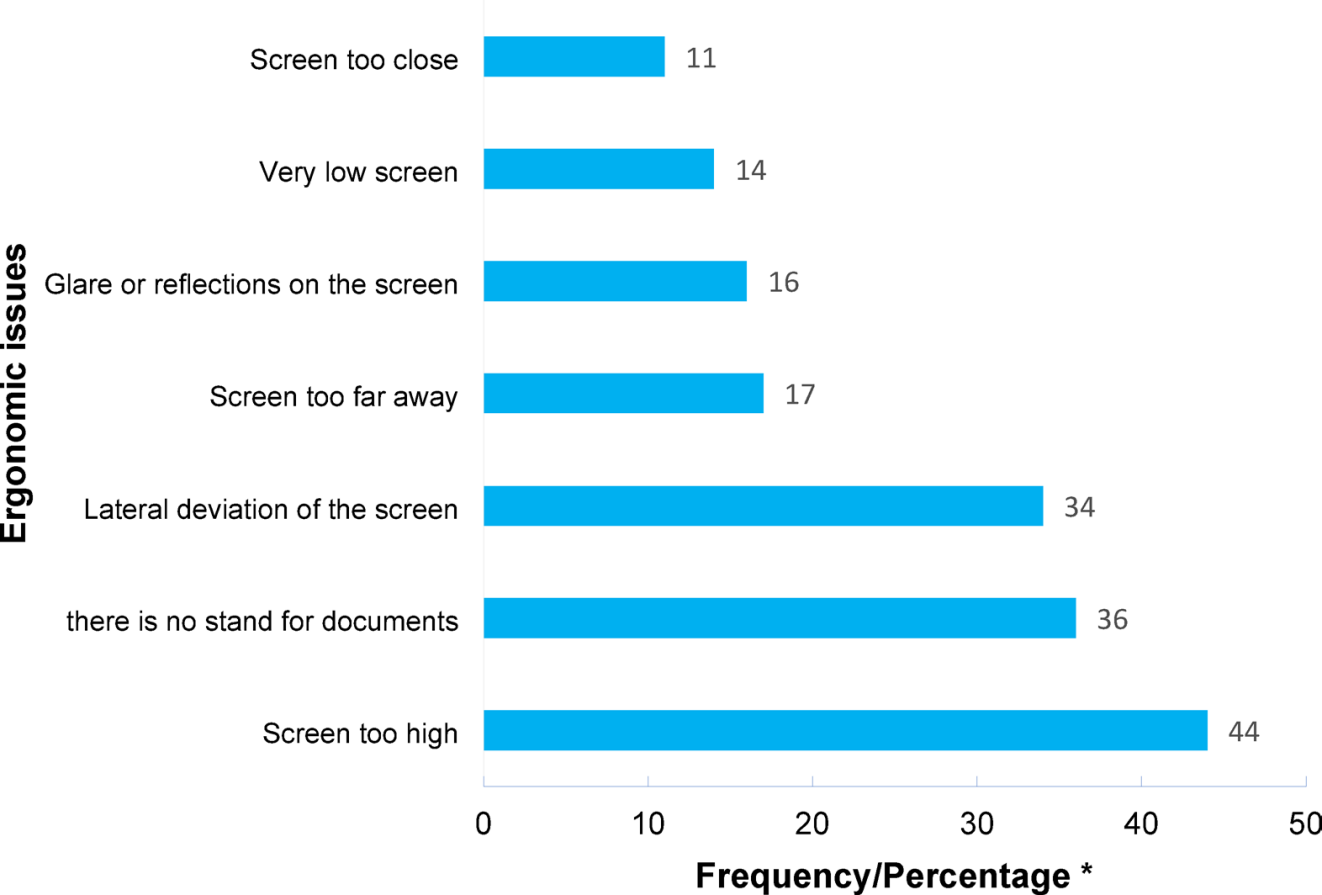
Frequency/percentage of observed ergonomic issues in monitor using the ROSA checklist. *Due to the sample size being close to 100, the frequency is approximately equal to the percentage value.

**Fig. 5:**
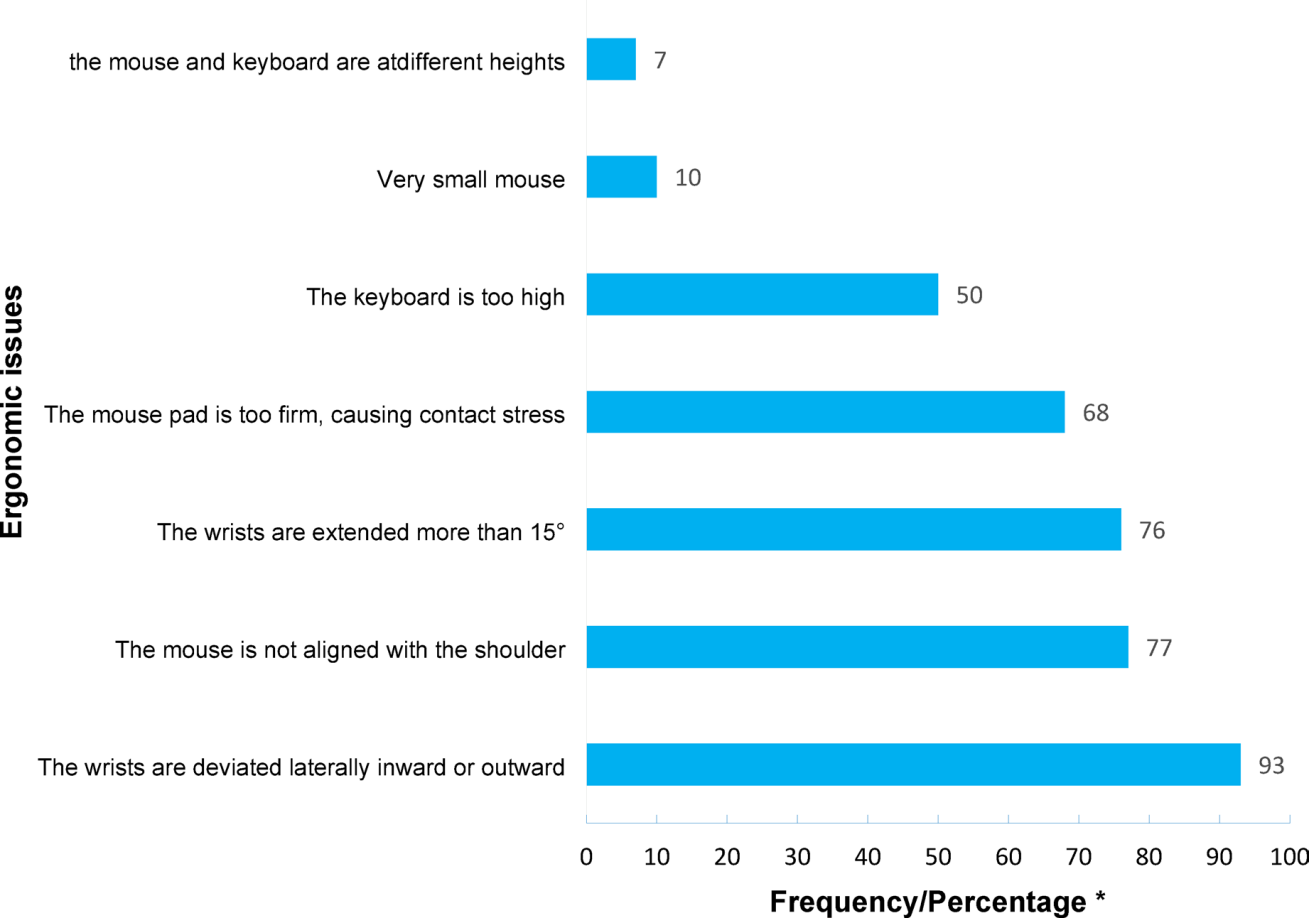
Frequency/percentage of observed ergonomic issues in mouse and keyboard using the ROSA checklist. *Due to the sample size being close to 100, the frequency is approximately equal to the percentage value.

The evaluation of 99 office workstations yielded a mean ROSA score of 5.40 (Standard Deviation = 1.27) on a 10-point scale. The mean scores for workstation components were: chair: 4.21 (1.42), monitor-telephone: 3.10 (1.65), and mouse-keyboard: 3.98 (1.31). Chairs were identified as the component posing the greatest physical strain. Notably, 54.5% of workers lacked desk phones and relied solely on mobile devices. The frequency of specific ergonomic issues for each workstation component is detailed in Figs. 3, 4 and 5. Time duration of use analysis revealed chairs had the highest utilization time among all workstation components. Complete temporal patterns for each component, presented as frequency counts and percentages, are available in Appendix 2.

### Associations between risk factors and MSDs symptoms

Independent t-tests revealed that participants with MSDs symptoms in the knees, shoulders, and upper back had significantly higher seat height scores than those without symptoms (*p* < 0.01). Significant associations were also found between the ROSA-B (composite score of monitor and telephone) and neck pain, and between the ROSA-A (composite score of chair items) and the ROSA-C (composite score of mouse and keyboard) and shoulder pain (*p* < 0.05). The final ROSA score showed significant associations with MSDs symptoms in the neck, shoulder, and back regions (*p* < 0.05) (Table 2).

**Table 2:** Results of independent t-tests examining differences between MSD symptoms in five high-prevalence body regions and ROSA checklist sub-scores.

ROSA sub-scores	Neck		Low-back		Sholders		Knees		Upper-back	
	Yes	No	Yes	No	Yes	No	Yes	No	Yes	No
	Mean (SD)	Mean (SD)	Mean (SD)	Mean (SD)	Mean (SD)	Mean (SD)	Mean (SD)	Mean (SD)	Mean (SD)	Mean (SD)
Seat height (1–5 points)	1.46 (0.94)	1.24 (0.70)	1.42 (0.96)	1.32 (0.72)	1.85 (1.16)^***^	1.12 (0.48)	1.70 (1.10)^**^	1.18 (0.59)	1.95 (1.02)^***^	1.22 (0.72)
Seat depth (1–3 points)	2.48 (0.84)	2.63 (0.80)	2.45 (0.80)	2.55 (0.85)	2.56 (0.75)	2.54 (0.87)	2.54 (0.80)	2.55 (0.88)	2.52 (0.75)	2.55 (0.85)
Armrests (1–5 points)	2.77 (0.84)	2.49 (1.03)	2.67 (0.83)	2.64 (1.03)	2.73 (0.99)	2.61 (0.90)	2.73 (0.84)	2.61 (0.98)	2.90 (0.94)	2.59 (0.92)
Backrest (1–4 points)	2.29 (0.99)	1.95 (0.77)	2.29 (0.85)	2.00 (0.98)	2.32 (0.88)	2.06 (0.93)	2.22 (0.95)	2.11 (0.91)	2.43 (1.03)	2.08 (0.88)
Monitor (1–7 points)	3.69 (1.73)^*^	2.93 (1.40)	3.65 (1.64)	3.06 (1.59)	3.56 (1.50)	3.28 (1.71)	3.67 (1.55)	3.19 (1.68)	3.43 (1.32)	3.36 (1.72)
Mouse (1–7 points)	2.74 (0.87)	2.66 (0.82)	2.67 (0.88)	2.74 (0.82)	2.50 (1.02)	2.81 (0.73)	2.84 (0.81)^*^	2.49 (0.87)	2.71 (0.84)	2.70 (0.85)
Keyboard (1–7 points)	3.05 (0.92)	3.14 (0.94)	3.19 (0.82)	2.98 (1.03)	3.23 (0.85)	3.01 (0.96)	3.19 (0.91)	3.03 (0.94)	3.48 (0.87)^*^	2.99 (0.92)
^a^ ROSA – A	4.50 (1.39)^*^	3.80 (1.36)	4.33 (1.32)	4.08 (1.51)	4.62 (1.50)^*^	4.00 (1.33)	4.51 (1.48)	4.03 (1.35)	4.95 (1.63)^**^	4.01 (1.29)
^b^ ROSA – B	3.50 (1.71)^**^	2.54 (1.40)	3.40 (1.71)^*^	2.77 (1.53)	3.41 (1.63)	2.94 (1.65)	3.40 (1.57)	2.92 (1.68)	3.19 (1.43)	3.08 (1.71)
^c^ ROSA – C	4.24 (1.27)^*^	3.63 (1.30)	4.06 (1.23)	3.91 (1.41)	4.32 (1.22)^*^	3.81 (1.33)	4.05 (1.29)	3.95 (1.33)	4.57 (1.16)^*^	3.83 (1.31)
Final ROSA Score	5.77 (1.20)^***^	4.88 (1.19)	5.44 (1.24)	5.36 (1.31)	5.82 (1.34)^*^	5.18 (1.18)	5.65 (1.42)	5.26 (1.16)	6.00 (1.52)^*^	5.24 (1.15)

^*^0.01 < p-value < 0.05.

^**^0.001 < p-value < 0.01.

^***^p-value < 0.001.

^a^ Composite score of chair height, depth, armrests, backrest, and usage time duration; ^b^Composite score of monitor, telephone, and their usage time duration; ^c^Composite score of mouse, keyboard, and their usage time duration.

As shown in Table 3, participants with MSDs symptoms in all five high-prevalence body regions reported significantly higher job stress than those without symptoms (*p* < 0.05). Significant associations were also observed between low back pain and increased age, work experience, and mobile phone use, while higher physical activity levels were associated with reduced low back pain (*p* < 0.05).

**Table 3:** Results of independent t-tests examining differences between MSDs symptoms in five high-prevalence body regions and individual/psychological risk factors (quantitative variables).

individual/psychological risk factors	Neck		Low-back		Sholders		Knees		Upper-back	
	Yes	No	Yes	No	Yes	No	Yes	No	Yes	No
Age (year)	34.66 (6.37)	34.07(5.07)	35.67 (6.12)^*^	33.02 (5.25)	34.88 (6.54)	34.17 (5.48)	34.76 (5.87)	34.21 (5.87)	34.62 (6.36)	34.36 (5.59)
Height (cm)	175.74 (9.39)	178.39 (9.29)	176.13 (9.2)	177.62 (9.6)	174.88 (9.1)	177.86 (9.4)	176.68 (8.6)	176.94 (9.9)	173.71 (9.6)	177.68 (9.2)
Weight (Kg)	82.62 (16.91)	88.22 (17.20)	85.69 (17.7)	84.11 (16. 7)	81.24 (17.8)	86.88 (16.6)	85.51 (17.3)	84.60 (17.2)	82.48 (15.8)	85.60 (17.5)
Body mass index (kg/m^2^)	26.59 (4.17)	27.57 (4.33)	27.43 (4.38)	26.52 (4.07)	27.33 (4.14)	26.36 (4.41)	27.19 (4.06)	26.87 (4.37)	27.20 (4.09)	26.94 (4.30)
Work experience (year)	9.21 (5.24)	9.73 (5.15)	10.81 (5.6)^**^	7.89 (4.24)	9.97 (5.87)	9.14 (4.81)	9.91 (5.35)	9.14 (5.10)	8.79 (5.71)	9.60 (5.06)
Job stress (1-100 points)	71.12 (19.4)^*^	59.63 (27.28)	72.8(19.6)^**^	59.25 (26.5)	76.6(17.9)^**^	61.00 (25.2)	75.0(16.3)^**^	61.21 (26.42)	79.0 (17.5)^**^	62.95 (24.5)
Personal life stress	43.71 (26.30)	37.80 (27.4)	42.98 (25.3)	39.36 (28.5)	40.73 (25.7)	41.54 (27.5)	42.30 (28.2)	40.64 (26.11)	53.81 (30.0)^*^	37.88 (24.0)
Mobile phone use (hours)	4.20 (2.32)	3.98 (1.64)	4.53 (2.08)^*^	3.82 (2.02)	4.20 (2.67)	4.06 (1.68)	4.17 (1.70)	4.07 (2.26)	3.95 (2.07)	4.15 (2.07)
Sedentary work per day(hours)	5.89 (1.47)^**^	5.02 (1.75)	5.82 (1.60)	5.21 (1.64)	5.96 (1.72)	5.31 (1.56)	6.00 (1.25)^*^	5.25 (1.78)	5.74 (1.64)	5.47 (1.64)
Exercise per week (hours)	2.89 (3.51)	3.76 (3.81)	2.28 (3.06)	4.32 (3.96)^**^	3.19 (3.31)	3.28 (3.83)	2.74 (3.36)	3.55 (3.80)	3.05 (3.46)	3.30 (3.71)

^*^0.01 < p-value < 0.05.

^**^0.001 < p-value < 0.01.

^***^p-value < 0.001.

Chi-square tests indicated that men experienced significantly more neck and shoulder pain than women (*p* < 0.05). Managers exhibited a significantly higher prevalence of lower back MSD symptoms compared to office staff (*p* < 0.05) (Table 4).

**Table 4:** Results of Chi-square tests examining differences between prevalence of MSDs symptoms in each body region and individual risk factors (qualitative variables).

Individual risk factors		Neck			Low-back			Sholders			Knees				Upper-back			
		Yes	No	p-value	Yes	No	p-value	Yes	No	p-value	Yes		No	p-value	Yes		No	p-value
		N (%)	N (%)		N (%)	N (%)		N (%)	N (%)		N (%)		N (%)		N (%)		N (%)	
Gender	Male	39 (39.4)	36 (36.4)	**0.031**	38 (38.4)	37 (37.4)	0.640	21 (21.2)	54 (54.5)	**0.026**	26 (26.3)		49 (49.5)	0.342	13 (13.1)		62 (62.6)	0.149
	Female	19 (19.2)	5 (5.1)		14 (14.1)	10 (10.1)		13 (13.1)	11 (11.1)		11 (11.1)		13 (13.1)		8 (8.1)		16 (16.2)	
Job position	Employee	33 (33.3)	26 (26.3)	0.540	25 (25.3)	34 (34.3)	**0.024**	20 (20.2)	39 (39.4)	0.910	18 (18.2)		41 (41.4)	0.095	13 (13.1)		46 (46.5)	0.808
	Manager	25 (25.3)	15 (15.2)		27 (27.3)	13 (13.1)		14 (14.1)	26 (26.3)		19 (19.2)		21 (21.2)		8 (8.1)		32 (32.3)	
Exercise adherence	Yes	25 (25.3)	24 (24.2)	0.156	21 (21.2)	28 (28.3)	0.071	19 (19.2)	30 (30.3)	0.402	16 (16.2)		33 (33.3)	0.408	11 (11.1)		38 (38.4)	0.810
	No	33 (33.3)	17 (17.2)		31 (31.3)	19 (19.2)		15 (15.2)	35 (35.4)		21 (21.2)		29 (29.3)		10 (10.1)		40 (40.4)	
Having a second job	Yes	13 (13.1)	11 (11.1)	0.641	12 (12.1)	12 (12.1)	0.818	9 (9.1)	15 (15.2)	0.806	9 (9.1)		15 (15.2)	0.988	7 (7.1)		17 (17.2)	0.389
	No	45 (45.5)	30 (30.3)		40 (40.4)	34 (35.4)		25 (25.3)	50 (50.5)		28 (28.3)		47 (47.5)		14 (14.1)		61 (61.6)	
Smoking	Yes	14 (14.1)	11 (11.1)	0.817	14 (14.1)	11 (11.1)	0.818	6 (6.1)	19 (19.2)	0.234	6 (6.1)		19 (19.2)	0.152	4 (4.0)		21 (21.2)	0.578
	No	44 (44.4)	30 (30.3)		38 (38.4)	36 (36.4)		28 (28.3)	46 (46.5)		31 (31.3)		43 (43.4)		17 (17.2)		57 (57.6)	
Marital status	Single/ Divorced	18 (18.2)	16 (16.2)	0.445	15 (15.1)	19 (19.2)	0.252	12 (12.1)	22 (22.2)	0.745	16 (16.2)		18 (18.2)	0.218	9 (9.1)		25 (25.2)	0.525
	Married	40 (40.4)	25 (25.3)		37 (37.4)	28 (28.3)		22 (22.2)	43 (43.4)		21 (21.2)		44 (44.4)		12 (12.1)		53 (53.5)	
Number of children	No children	29 (29.3)	24 (24.2)	0.481	25 (25.3)	28 (28.3)	0.208	20 (20.2)	33 (33.3)	0.652	20 (20.2)		33 (33.3)	0.945	11 (11.1)		42 (42.4)	0.831
	One	13 (13.1)	8 (8.1)		10 (10.1)	11 (11.1)		8 (8.1)	13 (13.1)		8 (8.1)		13 (13.1)		5 (5.1)		16 (16.2)	
	Two	14 (14.1)	8 (8.1)		15 (15.2)	7 (7.1)		5 (5.1)	17 (17.2)		8 (8.1)		14 (14.1)		4 (4.0)		18 (18.2)	
	More than two	2 (2.0)	1 (1.0)		2 (2.0)	1 (1.0)		1 (1.0)	2 (2.0)		1 (1.0)		2 (2.0)		1 (1.0)		2 (2.0)	

## Discussion

Our study identified a remarkably high prevalence of work-related musculoskeletal disorders (WMSDs) among office workers, affecting 80.81% of the cohort. The neck (58.6%), lower back (52.5%), and shoulders (37.4%) were the most commonly affected regions (Fig. 2), a finding consistent with reports from similar occupational settings^2,30,31^. Ergonomic assessments revealed that workstations posed a substantial risk, with a mean ROSA score of 5.40 (SD = 1.27) on the 10-point scale. This exceeds the established risk threshold of 5^25^, indicating that current workstation configurations may pose substantial ergonomic hazards. This result supports Sonne et al.‘s conclusion that inadequate workstation design is a key contributor to increased MSDs risk in office personnel^16^.

Our analysis revealed significantly higher seat height scores among participants with MSDs symptoms in the knees, shoulders, and upper back compared to asymptomatic individuals (Table 2). Furthermore, seat height issues—specifically, *seat height too high; non-adjustable seat height; seat height too low*, were the most frequently identified chair-related ergonomic problem (*n* = 19; Fig. 3). These findings strongly suggest that improper seat height is a major ergonomic risk factor for MSDs in these anatomical regions. This conclusion is supported by Fitrianto et al., who also identified non-adjustable seat height as a contributor to MSD development in office environments^32^.

As shown in Table 2, ergonomic issues related to monitor setup (ROSA–B), chair configuration (ROSA–A), and mouse/keyboard placement (ROSA–C) were associated with neck pain prevalence. A common factor among these components was usage duration, which is incorporated into the ROSA A, B, & C sub-scores. Figure 4 highlights that monitor-related issues—which, according to ergonomic principles, may contribute to neck MSDs—included *“Screen too high”*,* “there is no stand for documents”*,* “Lateral deviation of the screen” and “Very low screen”.* These issues were highly prevalent among participants. These findings align with Kim et al., who reported that improper monitor positioning (above or below eye level) increases neck pain risk^33^.

Regarding chair ergonomics, seat height adjustments (a frequently reported issue) were identified as a key contributor to neck pain. Thus, a significant proportion of neck pain cases may stem from these ergonomic deficiencies. However, the role of non-ergonomic risk factors—including " Job Stress”, “Sedentary work per day “, and “mobile phone use”—should not be overlooked, as they also showed significant associations with neck pain. These results support Liu et al., who demonstrated that combined ergonomic and psychosocial factors have a synergistic effect on neck pain development^34^. Additionally, Chen et al. confirmed that prolonged smartphone use significantly increases neck pain risk^35^.

The results showed a significant association between shoulder pain and increased composite scores of chair components (ROSA-A) and mouse-keyboard (ROSA-C) (Table 2). Given the high frequency of ergonomic problems related to mouse and keyboard (“The mouse is not aligned with the shoulder” and “The keyboard is too high”) and chair height, these problems contribute substantially to musculoskeletal disorders in the shoulder region (Figs. 3 and 5). However, job stress (Table 3), gender, and job position also contribute to problems in this region. According to the present study’s results, men in the workplace suffer significantly more from neck and shoulder pain than women, and the prevalence of musculoskeletal disorder symptoms in the lower back region was significantly higher among managers than office staff (Table 4). These gender- and job-related differences are consistent with the findings of Demissie et al., who reported that men and individuals in managerial positions are more susceptible to developing musculoskeletal disorders due to higher work pressure and longer sitting hours^36^. Scientific evidence indicates that job stress increases muscular tension in the shoulder region through psycho-neural mechanisms^37^.

The findings of the current study revealed a significant association between increased keyboard score and combined chair score (ROSA-A) recorded in the ROSA checklist with the prevalence of musculoskeletal symptoms in the back region (Table 2). From an ergonomic perspective, the key factors contributing to these disorders in workstations include flexion related to inappropriate keyboard or chair height and spinal torsion resulting from improper keyboard and other interface device positioning. According to the findings, these ergonomic problems were highly prevalent in the studied office workstations (Figs. 3 and 5). These results align with previous findings that reported a direct relationship between improper chair design and increased risk of low back pain^32^. The study by Guo et al. also confirms that inappropriate keyboard height increases spinal flexion and consequently exerts pressure on lumbar discs^38^. Furthermore, in the present study, a statistically significant association was observed between the Final ROSA Score and the occurrence of musculoskeletal disorder symptoms in the neck, shoulder, and back regions (Table 2), which substantiates the more prominent role of office workstation ergonomic problems in developing pain in these areas compared to other risk factors.

The results demonstrated a significant association between low back pain and increasing age as well as work experience (Table 3). However, among the ROSA checklist items, only the composite monitor score (ROSA-B) showed a significant relationship with the occurrence of low back pain (Table 2). Ergonomic issues related to monitor setup were also highly prevalent, likely confirming their contribution to low back pain among the studied office workers (Fig. 4). The current study, consistent with previous research^2,3,10,11,18,20,21,36,37,39^, confirms the multifactorial nature of musculoskeletal disorders, particularly in the low back region. In addition to physical factors associated with office workstations, demographic factors (age and work experience - Table 3), job stress (Table 3), and mobile phone use also played a role in low back pain occurrence, with statistically significant relationships observed for each factor.

Based on the findings, targeted interventions are recommended, including cost-effective engineering controls (e.g., monitor stands, keyboard/mouse trays, document holders) and ergonomic training to correct posture and workstation setup. Furthermore, prolonged sitting was significantly associated with increased neck and knee pain, reinforcing the need for initiatives that promote physical activity and regular breaks from sedentary behavior^40,41^. A key strength of this study is its integrated assessment using objective, validated tools (ROSA and NMQ-E) within a real-world industrial-office setting, providing a high-resolution, multifactorial analysis of WMSD risks that corroborates and extends models of their complex etiology^19,21,32^. However, the study’s limitations, including the use of a VAS scale for job stress and its cross-sectional design, necessitate future longitudinal studies with more comprehensive psychosocial instruments to establish causality. Future research should also investigate the cost-effectiveness of the proposed ergonomic interventions and explore these relationships in larger, more diverse occupational cohorts. For practice, our findings provide a clear evidence-based framework for organizations to implement combined ergonomic and psychosocial risk management programs, ultimately aiming to reduce the substantial burden of WMSDs in modern office environments.

## Conclusion

This study revealed a high prevalence of work-related musculoskeletal disorders (80.81%) among office workers, primarily affecting the neck, lower back, and shoulders. Ergonomic assessments using the ROSA method demonstrated that most workstations exceeded recommended risk thresholds, with chairs identified as the most problematic component. Significant associations were found between specific ergonomic factors (seat height, monitor positioning) and MSDs symptoms, while job stress emerged as a consistent psychosocial risk factor across all body regions. The study contributes to occupational health literature by providing comprehensive evidence on the multifactorial nature of MSDs in office settings and validating the application of ergonomic assessment tools in industrial administrative contexts. Future research should employ longitudinal designs to establish causal relationships, investigate intervention effectiveness across diverse office environments, and develop integrated assessment frameworks that simultaneously address physical, psychological, and organizational risk factors.

## Supplementary Information

Below is the link to the electronic supplementary material.

Supplementary Material 1

## Data Availability

The data that support the findings of this study are available upon request from the corresponding author.

## References

[1] Aghilinejad, M., Farshad, A., Mostafaei, M. & Ghafari, M. Occupational medicine practice. (Tehran: Arjmand Publication, 2001).

[2] Cho, C. Y., Hwang, Y. S. & Cherng, R. J. Musculoskeletal symptoms and associated risk factors among office workers with high workload computer use. *J. Manip. Physiol. Ther.* **35**, 534–540 (2012). 10.1016/j.jmpt.2012.07.004 22951267

[3] Grabara, M. Prevalence of musculoskeletal disorders among Polish white-collar workers: the role of physical activity and risk factors. *Front. Public. Health*. **13**, 1551728 (2025). 40260170 10.3389/fpubh.2025.1551728 PMC12010249

[4] Hokmabadi, R. & Fallah, H. Ergonomic assessment of musculoskeletal disorders risk factors in construction workers by PATH method. *J. North. Khorasan Univ. Med. Sci.* **5**, 55–61 (2013).

[5] Yu, W. et al. Effectiveness of participatory training for prevention of musculoskeletal disorders: a randomized controlled trial. *Int. Arch. Occup. Environ. Health*. **86**, 431–440 (2013). 22544420 10.1007/s00420-012-0775-3

[6] Fung, I. W. H., Tam, V. W. Y., Tam, C. M. & Wang, K. Frequency and continuity of work-related musculoskeletal symptoms for construction workers. *J. Civil Eng. Manag.* **14**, 183–187 (2008).

[7] Hagberg, M., Morgenstern, H. & Kelsh, M. Impact of occupations and job tasks on the prevalence of carpal tunnel syndrome. *Scandinavian J. Work Environ. & Health*, 337–345 (1992). 10.5271/sjweh.1564 1485158

[8] Meena, M., Dangayach, G. & Bharadwaj, A. *Proceedings of the International conference of Mechanical, production and Automobile Engineering.* 247–249.

[9] Obasi, I. C. & Benson, C. The impact of digitalization and information and communication technology on the nature and organization of work and the emerging challenges for occupational safety and health. *Int. J. Environ. Res. Public Health* **22**, 362 (2025). 40238361 10.3390/ijerph22030362 PMC11942091

[10] Amer, S. et al. Prevalence of musculoskeletal disorders and their associated risk factors among computer users. *Indonesian J. Occup. Saf. Health*. **14**, 56–66 (2025).

[11] Nejad, N. H., Mohammadian, M., Haghdoost, A. A. & Charkhloo, E. The relationship of grip and pinch strength to musculoskeletal disorders in female carpet weavers in southeastern Iran, 2019. *Indian J. Occup. Environ. Med.* **25**, 138–146. 10.4103/ijoem.IJOEM_223_20 (2021). 34759600 10.4103/ijoem.IJOEM_223_20 PMC8559880

[12] Lis, A. M., Black, K. M., Korn, H. & Nordin, M. Association between sitting and occupational LBP. *Eur. Spine J.* **16**, 283–298 (2007). 16736200 10.1007/s00586-006-0143-7 PMC2200681

[13] Thorp, A. A. et al. Prolonged sedentary time and physical activity in workplace and non-work contexts: a cross-sectional study of office, customer service and call centre employees. *Int. J. Behav. Nutr. Phys. Activity*. **9**, 1–9 (2012). 10.1186/1479-5868-9-128 PMC3546308 23101767

[14] Johanning, E. & Bruder, R. Low back disorders and dentistry: stress factors and ergonomic intervention. *Ergonomics Dent. Care Worker Wash. DC*, 355–373 (1998).

[15] Ali, A., Puspitasari, N. B., Analisis risiko ergonomi work from office pada pekerja human resources development pt indo tambang raya menggunakan metode rapid office strain assesment (ROSA). *Indust. Eng. Online J.* **14** (2025).

[16] Sonne, M., Villalta, D. L. & Andrews, D. M. Development and evaluation of an office ergonomic risk checklist: ROSA–Rapid office strain assessment. *Appl. Ergon.* **43**, 98–108 (2012). 21529772 10.1016/j.apergo.2011.03.008

[17] de Barros, F. C. et al. Usefulness of the rapid office strain assessment (ROSA) tool in detecting differences before and after an ergonomics intervention. *BMC Musculoskelet. Disord.* **23**, 526 (2022). 35655178 10.1186/s12891-022-05490-8 PMC9160176

[18] Bernard, B. P. & Putz-Anderson, V. Musculoskeletal disorders and workplace factors; a critical review of epidemiologic evidence for work-related musculoskeletal disorders of the neck, upper extremity, and low back. (1997).

[19] Soares, C. O. et al. Preventive factors against work-related musculoskeletal disorders: narrative review. *Revista Brasileira De Med. Do Trabalho*. **17**, 415 (2020). 10.5327/Z1679443520190360 PMC7195879 32368676

[20] Taib, M. F. B. M. *Work Stress and Musculoskeletal Disorders Symptoms: The Effect of Psychosocial and Psychological Risk Factors*, 서울대학교 대학원, (2016).

[21] Warren, N. Work stress and musculoskeletal disorder etiology: the relative roles of psychosocial and physical risk factors. *Work* **17**, 221–234 (2001). 12441601

[22] Alammari, M. A. et al. Psychosocial and occupational factors associated with low back pain among healthcare professionals: A systematic review. *Cureus* **17** (2025). 10.7759/cureus.77426 PMC11823482 39949447

[23] Peacock, L. L. *ASSE Professional Development Conference and Exposition* ASSE-17-630 (2017).

[24] Mokhtarinia, H. R., Hosseini, Z. S. J., Shokouhyan, S. M. & Gabel, C. P. Translation, cultural adaptation and assessment of psychometrics properties of the extended version of the nordic musculoskeletal questionnaire (NMQ-E) in Persian Language speaking people. *BMC Musculoskelet. Disord.* **25**, 89 (2024). 38263054 10.1186/s12891-024-07192-9 PMC10804467

[25] Rodrigues, M. S. A. et al. Rapid office strain assessment (ROSA): cross cultural validity, reliability and structural validity of the Brazilian-Portuguese version. *Appl. Ergon.* **75**, 143–154 (2019). 30509519 10.1016/j.apergo.2018.09.009

[26] Armal, A., Mokhtarinia, H., Biglarian, A. & Abdi, K. Face and convergent validity of Persian version of rapid office strain assessment (ROSA) checklist. *Archives Rehabilitation*. **16**, 356–365 (2016).

[27] Boonstra, A. M., Preuper, S., Reneman, H. R., Posthumus, M. F., Stewart, R. E. & J. B. & Reliability and validity of the visual analogue scale for disability in patients with chronic musculoskeletal pain. *Int. J. Rehabil. Res.* **31**, 165–169. 10.1097/MRR.0b013e3282fc0f93 (2008). 18467932 10.1097/MRR.0b013e3282fc0f93

[28] Carlsson, A. M. Assessment of chronic pain. I. Aspects of the reliability and validity of the visual analogue scale. *Pain* **16**, 87–101. 10.1016/0304-3959(83)90088-X (1983). 6602967 10.1016/0304-3959(83)90088-X

[29] Manole, L. M. et al. Bioelectrical impedance analysis versus dual X-Ray absorptiometry for obesity assessment in pediatric populations: A systematic review. *Diagnostics* **15**, 1505 (2025). 40564826 10.3390/diagnostics15121505 PMC12192207

[30] Alaca, N., Acar, A. Ö. & Öztürk, S. Low back pain and sitting time, posture and behavior in office workers: A scoping review. *J. Back Musculoskelet. Rehabilit.* 10538127251320320 (2025). 10.1177/10538127251320320 40111906

[31] NODOUSHAN, R. J. et al. Investigating the prevalence of musculoskeletal disorders and degree of body discomfort in the office staff of the school of public Health, Shahid Sadoughi university of medical Sciences, Yazd. *Occupat. Hygiene Health Promot.* (2021).

[32] Fitrianto, T. R. et al. Evaluation of office chairs to enhance comfort and reduce musculoskeletal risk in sedentary work environments. *J. SENOPATI: Sustain. Ergon. Optim. Applic. Indust. Eng.* **6**, 114–121 (2025).

[33] Kim, H. et al. Comparison of neck pain and posture with spine angle tracking system between static and dynamic computer monitor use. *Electronics* **13**, 1363 (2024).

[34] Liu, F. et al. Mixed adverse ergonomic factors exposure in relation to work-related musculoskeletal disorders: a multicenter cross-sectional study of Chinese medical personnel. *Sci. Rep.* **15**, 1–12 (2025). 40289235 10.1038/s41598-025-99477-9 PMC12034785

[35] Chen, Y. J. et al. Association of smartphone overuse and neck pain: a systematic review and meta-analysis. *Postgrad. Med. J.*, qgae200 (2025). 10.1093/postmj/qgae200 39764644

[36] Demissie, B., Bayih, E. T. & Demmelash, A. A. A systematic review of work-related musculoskeletal disorders and risk factors among computer users. *Heliyon* **10** (2024). 10.1016/j.heliyon.2024.e25075 PMC10840111 38318034

[37] Alencar, M., da Silva, N. R. & Serranheira, F. d. C. B. d., Musculoskeletal pain and risk factors in office workers versus teleworkers: A systematic review. *Work* **80**, 974–997 (2025). 10.1177/10519815241289675 40297886

[38] Guo, Z. et al. Effects of laptop screen height on neck and shoulder muscle fatigue and spine loading for office workers. *Work* **79**, 1925–1937 (2024). 38995755 10.3233/WOR-230719

[39] Koukamari, P. H., Nikbakht, R., Karimy, M. & Mohammadi, Z. Psychosocial factors and musculoskeletal symptoms in office workers: validating the Maastricht upper extremity questionnaire. *Med. J. Islamic Repub. Iran.* **38**, 29 (2024). 10.47176/mjiri.38.29 PMC11129282 38803388

[40] Lin, J. H., Kaufman, B., Lin, R. T. & Bao, S. Sedentary behavior and musculoskeletal symptoms among work from home employees. *Int. J. Ind. Ergon.* **104**, 103653 (2024).

[41] Tersa-Miralles, C. et al. Effectiveness of workplace exercise interventions in the treatment of musculoskeletal disorders in office workers: a systematic review. *BMJ Open* **12**, e054288 (2022). 35105632 10.1136/bmjopen-2021-054288 PMC8804637

