# Supplementary material for: Musculoskeletal disorders among office workers: prevalence, ergonomic risk factors, and their interrelationships
Source: Sci Rep. 2025 Nov 26;15:45425. doi: 10.1038/s41598-025-30155-6 (PMC12749865; doi:10.1038/s41598-025-30155-6)
Supplement: Supplementary file 1 — Supplementary Material 1 [file 41598_2025_30155_MOESM1_ESM.docx]

**Appendices**

Appendix 1: Frequency (percentage) of positive responses to the Nordic Questionnaire items (indicating the presence of musculoskeletal disorder symptoms) among office workers

| **Body Regions** | Have you ever been hospitalized because of the trouble? | Have you ever had to change jobs or duties (even temporarily) because of the trouble? | Have you trouble (ache, pain or discomfort) at any time during the last month (4 weeks)? | Have you trouble (ache, pain or discomfort) at any time during the last 12 months? | **During the last 12 months have you at any time:** | | | |  |
| --- | --- | --- | --- | --- | --- | --- | --- | --- | --- |
|  |  |  |  |  | been prevented from doing your normal work (at home or away from home) because of the trouble? | seen a doctor, physiotherapist, chiropractor or other such person because of the trouble? | taken medication because of the trouble? | taken sick leave from work/studies because of the trouble? | |
| **Neck** | 5 (5.10) | 3 (3.00) | 58 (58.60) | 42 (42.40) | 14 (14.10) | 19 (19.20) | 19 (19.20) | 5 (5.10) |  |
| **Shoulders** | 0 (0.00) | 0 (0.00) | 34 (34.30) | 25 (25.30) | 7 (7.10) | 12 (12.10) | 12 (12.10) | 2 (2.00) |  |
| **Upper back** | 0 (0.00) | 0 (0.00) | 21 (21.20) | 5 (5.10) | 3 (3.00) | 3 (3.00) | 3 (3.00) | 2 (2.00) |  |
| **Elbows** | 0 (0.00) | 0 (0.00) | 16 (16.20) | 11 (11.10) | 3 (3.00) | 4 (4.00) | 4 (4.00) | 1 (1.00) |  |
| **Wrists/Hands** | 0 (0.00) | 0 (0.00) | 5 (5.10) | 17 (17.20) | 3 (3.00) | 3 (3.00) | 3 (3.00) | 2 (2.00) |  |
| **Low back** | 14 (14.10) | 7 (7.10) | 52 (52.50) | 43 (43.40) | 18 (18.20) | 20 (20.20) | 20 (20.20) | 14 (14.10) |  |
| **Hips/Thighs** | 0 (0.00) | 0 (0.00) | 7 (7.10) | 6 (6.10) | 3 (3.00) | 3 (3.00) | 3 (3.00) | 2 (2.00) |  |
| **Knees** | 8 (8.10) | 3 (3.00) | 37 (37.40) | 27 (27.30) | 8 (8.10) | 18 (18.20) | 18 (18.20) | 8 (8.10) |  |
| **Ankles/Feet** | 0 (0.00) | 0 (0.00) | 15 (15.20) | 11 (11.10) | 4 (4.00) | 7 (7.10) | 7 (7.10) | 1 (1.00) |  |

***
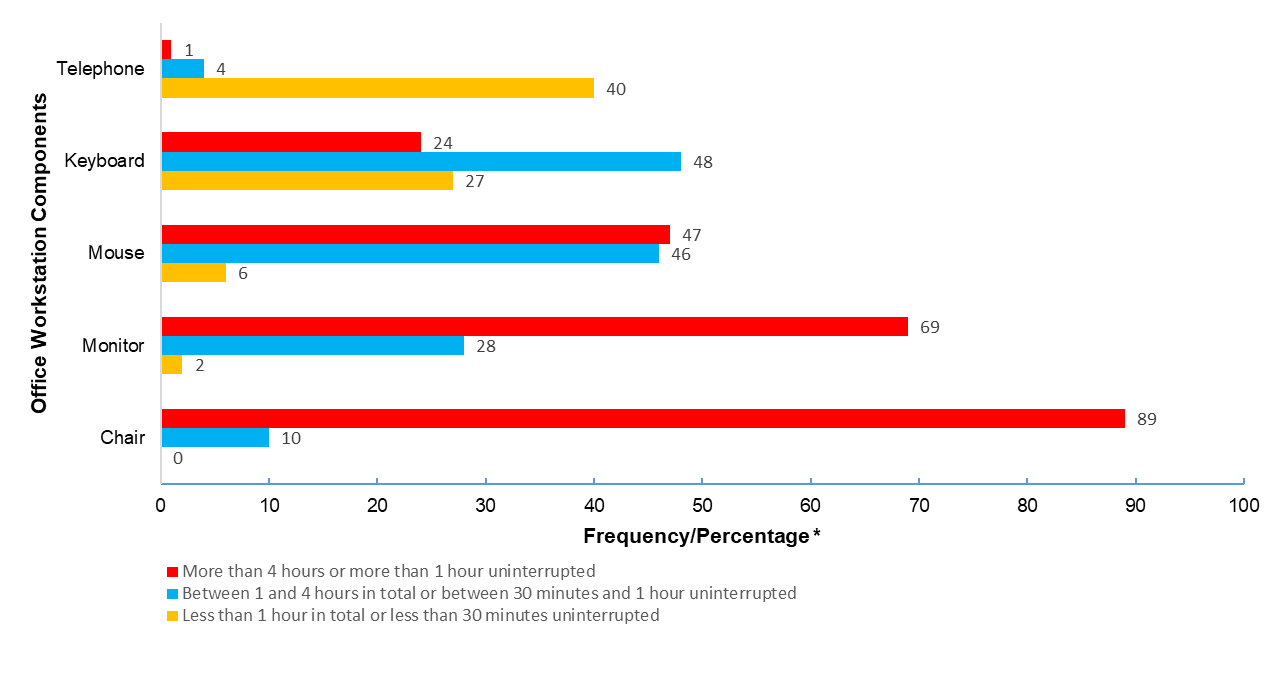
***

Appendix 2: Frequency/percentage of time duration of use for each component of the office workstation among studied administrative staff (n=99)

*Due to the sample size being close to 100, the frequency is approximately equal to the percentage value
